# *In Vivo* Dynamical Interactions between CD4 Tregs, CD8 Tregs and CD4^+^CD25^−^ Cells in Mice

**DOI:** 10.1371/journal.pone.0008447

**Published:** 2009-12-24

**Authors:** Arnon Arazi, Amir Sharabi, Heidy Zinger, Edna Mozes, Avidan U. Neumann

**Affiliations:** 1 Faculty of Life Sciences, Bar-Ilan University, Ramat Gan, Israel; 2 Department of Immunology, Weizmann Institute of Science, Rehovot, Israel; New York University, United States of America

## Abstract

**Background:**

Regulatory T cells (Tregs) were shown to be central in maintaining immunological homeostasis and preventing the development of autoimmune diseases. Several subsets of Tregs have been identified to date; however, the dynamics of the interactions between these subsets, and their implications on their regulatory functions are yet to be elucidated.

**Methodology/Principal Findings:**

We employed a combination of mathematical modeling and frequent *in vivo* measurements of several T cell subsets. Healthy BALB/c mice received a single injection of either hCDR1 - a tolerogenic peptide previously shown to induce Tregs, a control peptide or vehicle alone, and were monitored for 16 days. During this period, splenocytes from the treated mice were analyzed for the levels of CD4, CD25, CD8, CD28 and Foxp3. The collected data were then fitted to mathematical models, in order to test competing hypotheses regarding the interactions between the followed T cell subsets. In all 3 treatment groups, a significant, lasting, non-random perturbation of the immune system could be observed. Our analysis predicted the emergence of functional CD4 Tregs based on inverse oscillations of the latter and CD4^+^CD25^−^ cells. Furthermore, CD4 Tregs seemed to require a sufficiently high level of CD8 Tregs in order to become functional, while conversion was unlikely to be their major source. Our results indicated in addition that Foxp3 is not a sufficient marker for regulatory activity.

**Conclusions/Significance:**

In this work, we unraveled the dynamics of the interplay between CD4, CD8 Tregs and effector T cells, using, for the first time, a mathematical-mechanistic perspective in the analysis of Treg kinetics. Furthermore, the results obtained from this interdisciplinary approach supported the notion that CD4 Tregs need to interact with CD8 Tregs in order to become functional. Finally, we generated predictions regarding the time-dependent function of Tregs, which can be further tested empirically in future work.

## Introduction

Regulatory T cells play an important role in both health and disease, preventing the development of autoimmunity and regulating the normal immune response to invading pathogens [1]. Deficiencies in such cells have been associated with several autoimmune diseases [2], while their upregulation has been shown to be a key factor mediating the beneficial effects of novel experimental treatments to such diseases [3]–[5]. Several subsets of regulatory T cells have been identified to date [6]; however, their developmental dynamics, as well as the nature of interactions between them, are yet to be characterized.

A peptide, hCDR1, that is based on the sequence of the complementarity determining region (CDR)-1 of an autoantibody [7], was shown to ameliorate the serological and clinical manifestations of the autoimmune disease, systemic lupus erythematosus (SLE) [8]. The beneficial effects of hCDR1, following tolerogenic administrations, were demonstrated to be mediated via the induction of functional CD4^+^CD25^+^Foxp3^+^ regulatory T cells (CD4 Tregs) [4]. Furthermore, CD8^+^CD28^−^Foxp3^+^ cells (CD8 Tregs) play an important role in the ameliorative effects of hCDR1 as well, and were shown to be required for the optimal development and function of CD4 Tregs [9]. Moreover, a single injection of hCDR1 into healthy, naïve mice was also shown to induce functional CD4 Tregs capable of suppressing the activity of effector T cells, as demonstrated by the clinical improvement of SLE-afflicted mice administered with these cells [4], [9]. Thus, based on these results, it was of interest to study the interactions between these different cell subsets in healthy mice injected with hCDR1.

The application of mathematical models, in conjunction with kinetically-measured experimental and clinical data, has proven in the past to be an extremely useful approach, in particular in the fields of virology and immunology [10]–[12]. In addition to generally shedding light on the time-dependant behavior of the system at hand, such a methodology can produce both quantitative and qualitative insights into the underlying mechanisms [13], [14]. The kinetics of regulatory T cells have been studied in recent years [15]–[23]. However, this has not been yet done with regard to a non-immunogenic (tolerogenic) immunomodulation by a peptide. In addition, the interactions between different subsets of regulatory T cells have not been previously studied kinetically. While mathematical models have been applied to the investigation of Tregs dynamics by Vukmanovic-Stejic *et al.*, 2006, these models were merely *descriptive*, and did not incorporate an explicit specification of the biological *interactions* between different cell populations.

The objective of the present work has been to quantitatively characterize the time-dependent interplay between several immune subpopulations, and in particular CD4^+^CD25^−^, CD4^+^CD25^+^Foxp3^+^ and CD8^+^CD28^−^Foxp3^+^ cells, under tolerogenic conditions. To this end, the kinetics of the latter cell subsets were determined following a single subcutaneous injection of healthy mice with hCDR1. By fitting the measured biological data to mathematical models, the interactions between the 3 subpopulations were analyzed.

## Results

### *In Vivo* Kinetics of Different Spleen-Derived T Cell Subsets

**Figure 1:**
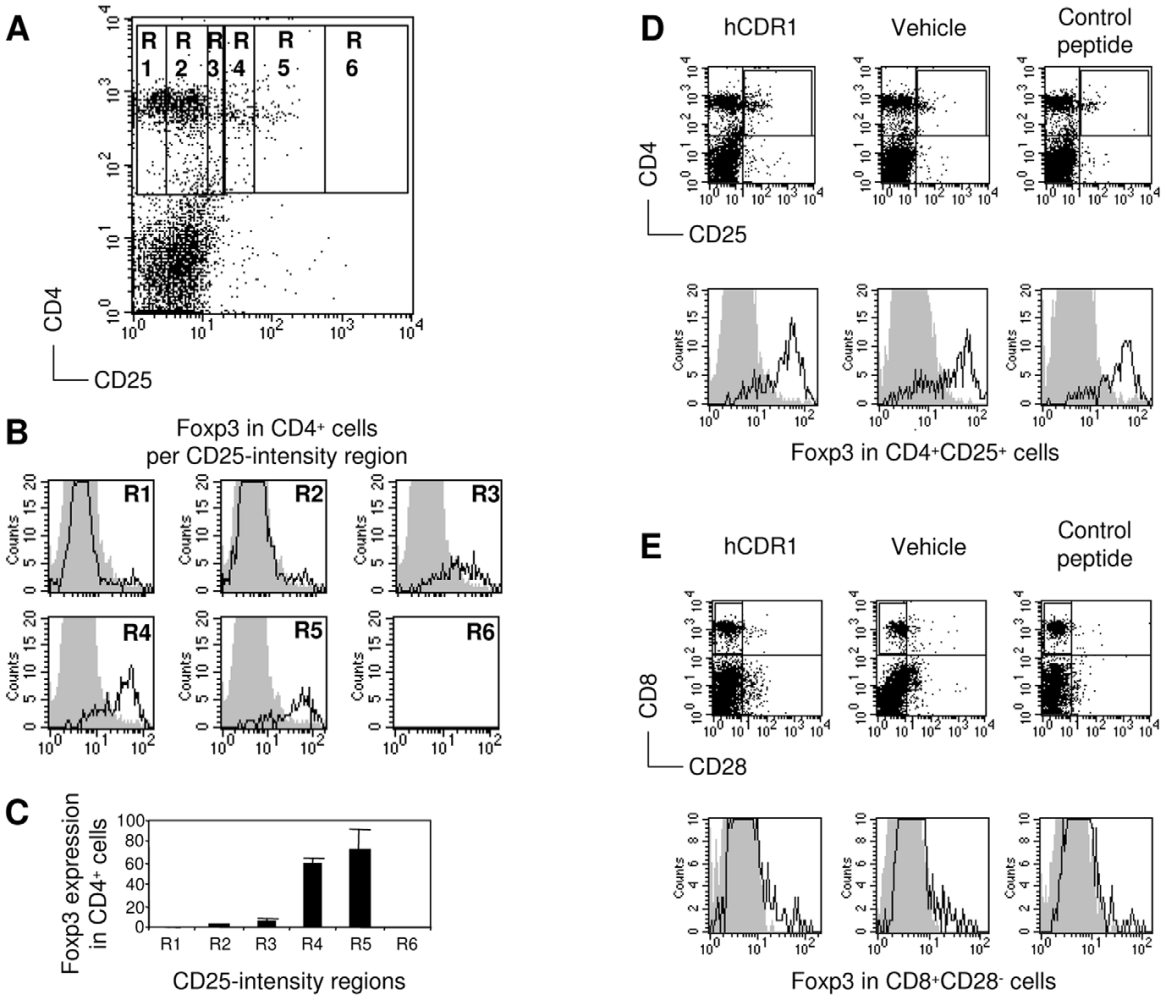
Plot settings for analyzing CD4 and CD8 Tregs by flow cytometry. Percentages of CD4^+^CD25^+^Foxp3^+^ cells (i.e. CD4 Tregs) and CD8^+^CD28^−^Foxp3^+^ cells in spleens from individual healthy BALB/c mice were determined at different time points over a period of 16 days following a single subcutaneous injection of either a tolerogenic peptide (hCDR1), a control peptide or vehicle alone. Spleen-derived cells were triple-stained with CD4, CD25 and Foxp3 and analyzed by flow cytometry. Shown are representative results obtained on day 9 following the injection. (**A**) The gate of CD4^+^ cells was subdivided for 6 regions corresponding to different intensities of staining with CD25. (**B**) The expression of Foxp3 was determined in the different regions. Gray contours indicate staining with the isotype control. (**C**) Foxp3 relative expression in CD4^+^ cells per intensity region of CD25 staining. Accordingly, cutting borders that summate regions R4, R5, and R6 of CD25 intensity should properly indicate regulatory phenotype based on Foxp3 staining.(**A**, **B**, **C**) present results with cells of mice injected with hCDR1 only. (**D**) Representative dot plots of CD4 and CD25-expressing spleen-derived cells and histograms of Foxp3 expression in the CD4^+^CD25^+^ cells from the 3 treatment groups. (**E**) Representative dot plots of CD8 and CD28-expressing spleen-derived cells and histograms of Foxp3 expression in the CD8^+^CD28^−^ cells from the 3 treatment groups.

Healthy BALB/c mice were divided into 3 treatment groups, receiving a single subcutaneous injection of either hCDR1, a control (scrambled) peptide, or vehicle alone. At each time point studied, 2 mice were sacrificed out of each treatment group, and the percentage of spleen-derived cells bearing the markers CD4, CD8, CD25, CD28 and Foxp3 was determined following staining with the relevant antibodies, using flow cytometry. Figure 1 demonstrates the analysis performed on a representative day.

**Figure 2:**
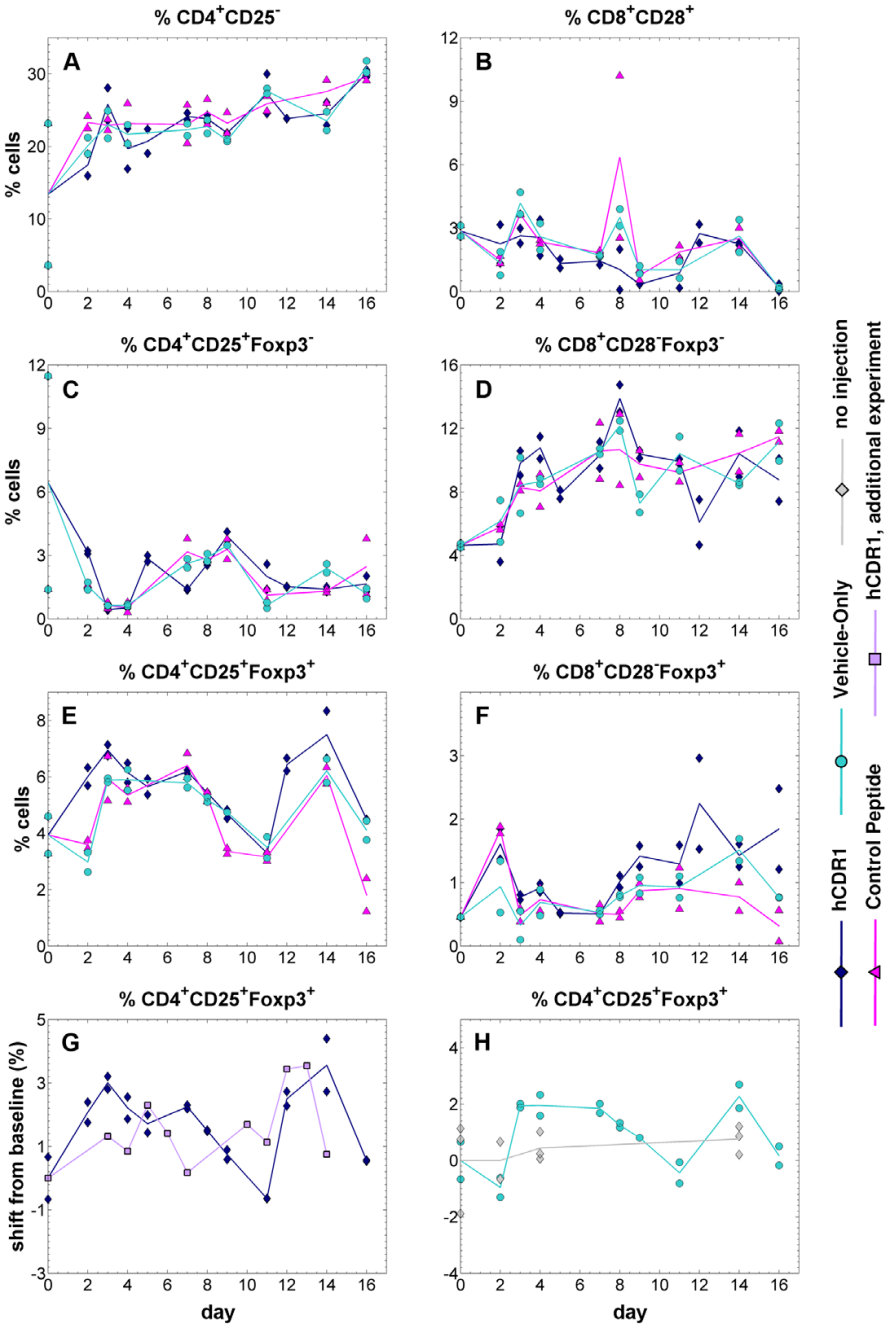
The kinetics of spleen-derived T cell subsets. Healthy BALB/c mice received a single injection of one of three alternative treatments (hCDR1, vehicle-only, control peptide). In each inspected day, 2 mice were sacrificed out of each treatment group, and the percentage of cells bearing the markers CD4, CD8, CD25, CD28 and Foxp3 was determined using flow cytometry. Points represent measured values, lines connect the daily averages. Presented cell subpopulations: (**A**) CD4^+^CD25^−^ (**B**) CD8^+^CD28^+^ (**C**) CD4^+^CD25^+^Foxp3^−^ (**D**) CD8^+^CD28^−^Foxp3^−^ (**E**) CD4^+^CD25^+^Foxp3^+^ (**F**) CD8^+^CD28^−^Foxp3^+^. Panel (**G**) compares the kinetics of CD4^+^CD25^+^Foxp3^+^ cells in two different experiments, following injection of hCDR1. Panel (**H**) presents measurements of CD4^+^CD25^+^Foxp3^+^ cells, taken from mice which received no injection, as compared to mice injected with vehicle alone. Note that both in panel (**G**) and panel (**H**), the reported data are absolute shifts from the baseline (i.e. day 0 measurements), to enable comparison over different experiments.

**Figure 3:**
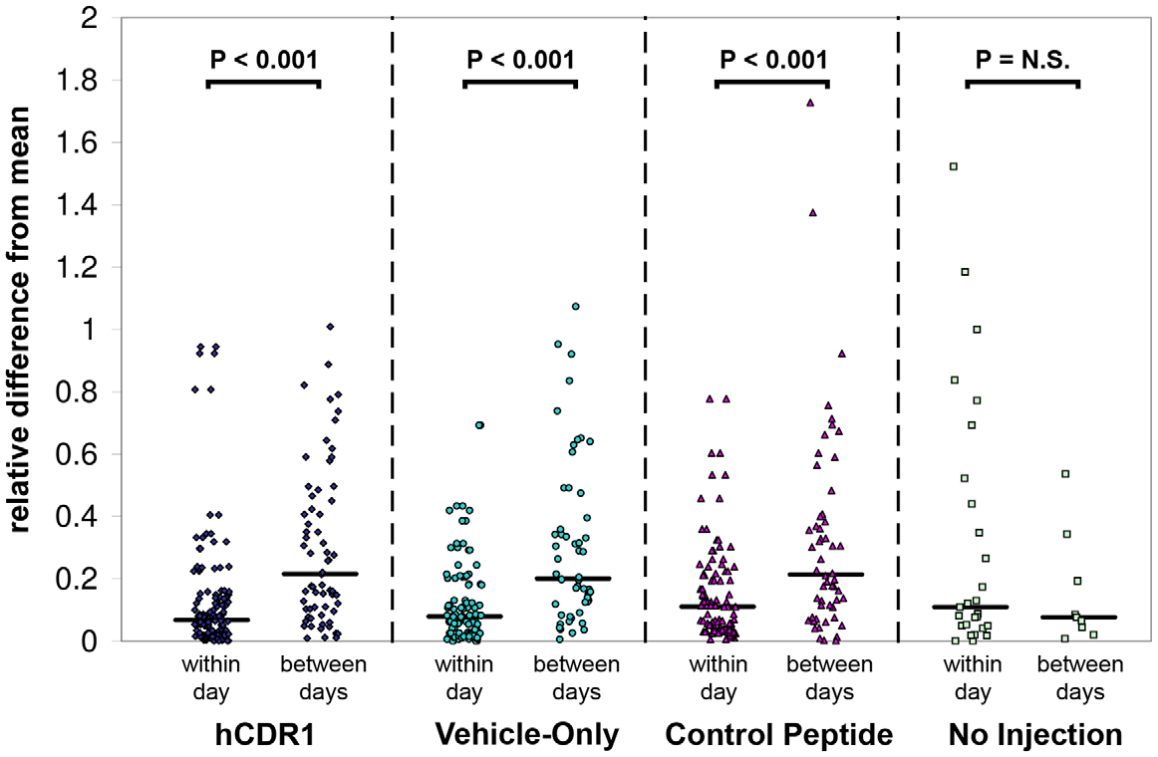
A comparison of the within-day and between-days variations for the different groups. The within-day variation was computed as the difference between each FACS measurement and its corresponding daily average (i.e. the average of the 2 measurements taken for the relevant day, cell subset and treatment group), divided by this average. The between-days variation was computed as the difference between each daily average and the overall average of the series (i.e., the average of all the measurements related to a cell subset and treatment group), divided by the overall average. The division by averages serves as a normalization step, allowing to compare all cell subsets together. Horizontal lines represent the medians. The statistical significance of the differences between the within-day and between-days variations was estimated using the non-parametric Mann-Whitney U-test, resulting with P<0.001 for each of the 3 treatment groups. For the non-injected mice, the difference between the within-day and between-days variations was not statistically significant.

Substantial changes in the *in vivo* levels of the studied cell populations were discernable over a period of 16 days following either injection (Figure 2). In all 3 treatment groups, an initial transient rise occurred in all cell subsets (with the exception of CD4^+^CD25^+^Foxp3^−^ cells) between days 0–4. Subsequently, the observed kinetic patterns demonstrated statistically significant cross-correlations between pairs of the different treatment groups for most of the cell subsets (see details in Table S1). This implies that the measured changes in cell populations did not represent mere stochastic fluctuations but rather a genuine biological process, since in the former case, the corresponding pairs of time series would not be expected to be correlated across time. Additional support for this statement is provided by the statistically significant difference between the relatively small variance of within-day measurements, as compared to the magnitude of between-days changes (Figure 3; p-value<0.001 for all treatment groups, Mann-Whitney U-test). Again, if the observed kinetics represented solely random fluctuations, the variance of within-day measurements and between-days changes would be expected to be similar – as was indeed the case for untreated mice (i.e. mice that were not injected with any substance) monitored over time. These observations diminish the potential uncertainty in the results due to the limited number of mice sacrificed each day in each treatment group. It is noteworthy that the vehicle-only injection produced kinetic patterns *qualitatively* similar to those obtained following both other types of treatment.

Quantitatively, the most pronounced differences between the kinetics induced by the 3 types of treatment were with regard to the CD4 and CD8 Foxp3-expressing T cells (Figure 2E–F). More specifically, these cells rose higher and earlier following the administration of hCDR1, compared to the other types of injections (days 7–11). Indeed, these cells have been previously shown to mediate the beneficial therapeutic effects of hCDR1 [4], [9]. The effect of hCDR1 was qualitatively reproduced in 3 independent experiments, with a total of N = 136 mice analyzed. As can be seen in Figure 2G, showing kinetic measurements of an additional experiment, some quantitative differences may exist between such repetitions; in particular, the exact timing of the rise in the level of CD4^+^CD25^+^Foxp3^+^ cells may slightly differ. In untreated mice the level of CD4^+^CD25^+^Foxp3^+^ remained more or less constant over time (Fig 2H).

Throughout this work, we referred in our analysis to measurements of cell percentages (out of total viable splenocytes, as obtained by FACS measurements) rather than absolute numbers of cells. This was done due to the additional noise introduced in the latter as a result of differences in spleen sizes and cell numbers between individual mice. Nonetheless, as exemplified in Figure S1, compared to Figure 2E, we verified that absolute cell numbers yielded qualitatively similar results to those reported here.

### Correlation between the Kinetics of CD4^+^CD25^−^, CD4^+^CD25^+^Foxp3^+^ and CD8^+^CD28^−^Foxp3^+^ Cells

**Figure 4:**
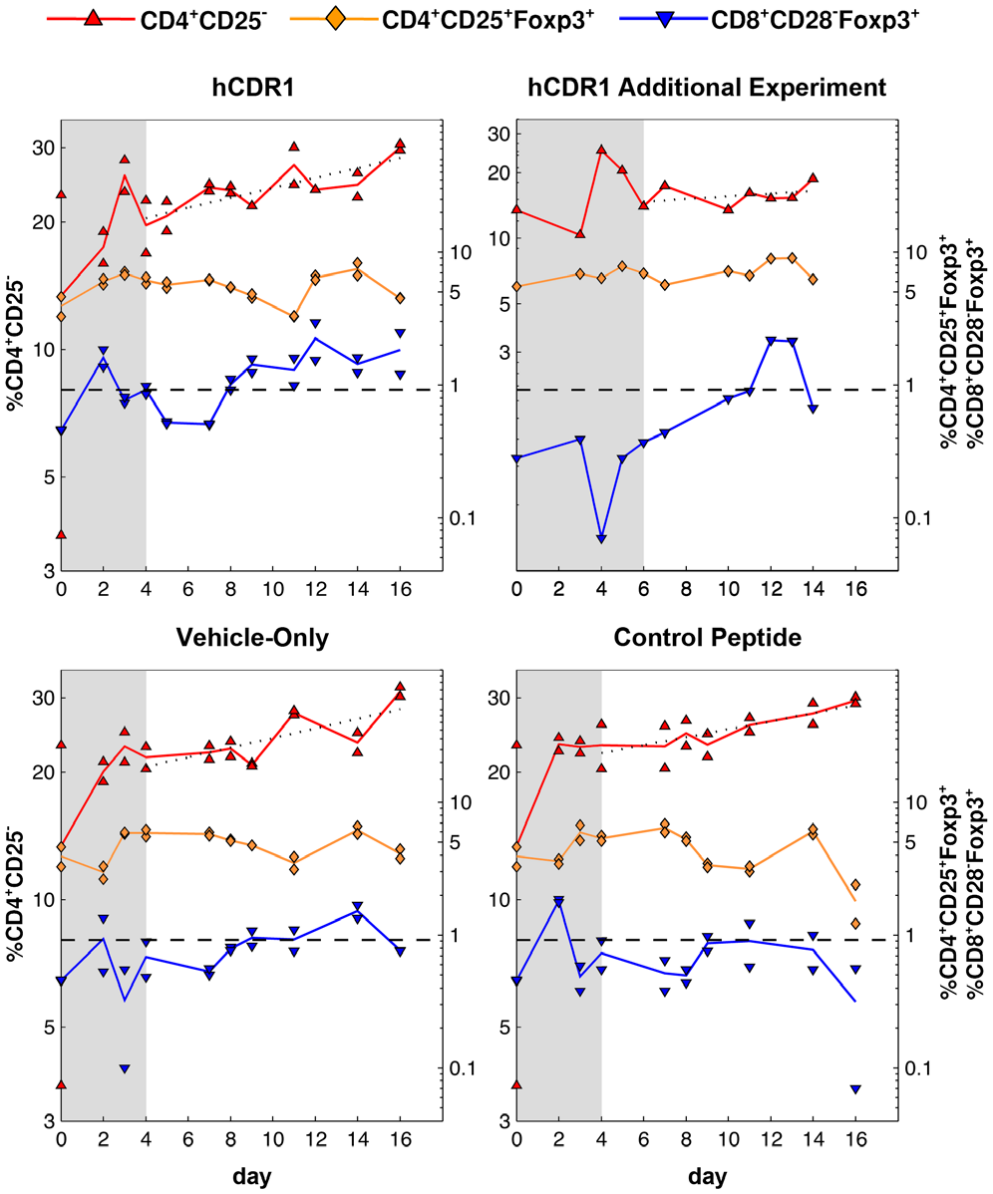
The correlated kinetics of CD4^+^CD25^−^, CD4^+^CD25^+^Foxp3^+^ and CD8^+^CD28^−^Foxp3^+^ cells, for each of the treatment groups. The levels of CD4^+^CD25^−^ (red) cells are specified on the left y-axis, those of CD4^+^CD25^+^Foxp3^+^ (orange) and CD8^+^CD28^−^Foxp3^+^ (blue) cells on the right y-axis. Points represent the values measured by FACS, lines connect daily averages. Note the logarithmic scale of both y-axes. Following an initial transient response in all 3 subsets (days 0–4; days 0–6 for the additional experiment involving the hCDR1 treatment), the CD4^+^CD25^−^ population grew in an approximately exponential process (dotted line; log-linear regression was calculated based on the measurements taken in the days following the initial response, i.e. disregarding the shaded areas). In the second part of the experiment (day 9 on) fluctuations of CD4^+^CD25^−^ cells around the regression line were inversely correlated with changes in CD4^+^CD25^+^Foxp3^+^ cell levels – but only in the hCDR1 and vehicle-only treatment groups, where CD8^+^CD28^−^Foxp3^+^ cells rose and persistently stayed above a hypothetical threshold (dashed line).

In all 3 treatment groups, there was an apparent and persistent growth in the population of CD4^+^CD25^−^ cells, with an overall increase of 124%, 120%, and 131% in the level of these cells following the hCDR1, control peptide, and vehicle injections, respectively. After the initial response (days 0–4, see above), these cells climbed gradually in an approximately exponential process (i.e., linear on a logarithmic scale; see dotted line in Figure 4), with similar doubling times in all 3 arms, equal to 26, 32, and 27 days. Moreover, during the later stages of this process (days 9–16), the CD4^+^CD25^−^ cells demonstrated fluctuations around the growth trend line which were inversely correlated with the kinetics of Foxp3-expressing CD4^+^CD25^+^ regulatory T cells; that is, a rise in the regulatory T cells corresponded to a decrease in the growth rate of CD4^+^CD25^−^ cells, and vice versa.

It is interesting to note that this inverse correlation was observable only after the late rise of CD8^+^CD28^−^Foxp3^+^ cells, in days 7–8 (Figure 4). Indeed, as our data show, a minor increase in the latter cells to a level lower than 1% corresponded to no apparent consequent fluctuations of the CD4^+^CD25^−^ cells, as was the case for the control peptide injection, compared with the other two treatment groups (Figure 4).

### Fitting the *In Vivo* Kinetics Using Mathematical Models

By fitting a mathematical model to the observed data, we were able to show here that the hypothesis that CD4^+^CD25^+^Foxp3^+^ cells modulate the proliferation rate of CD4^+^CD25^−^ cells after interacting with CD8^+^CD28^−^Foxp3^+^ cells matches well the measured kinetics. More specifically, the mathematical model used here represents 2 main biological assumptions: First, that CD4^+^CD25^+^Foxp3^+^ cells may expand independently (it should be noted that it is known that regulatory T cells, although usually anergic, may nonetheless expand in the proper milieu of cytokines). Second, that a rise in these cells reduces the proliferation rate of CD4^+^CD25^−^ cells and as a consequence their level (and vice versa) – but only if the CD4^+^CD25^+^Foxp3^+^ cells interacted first with CD8^+^CD28^−^Foxp3^+^ cells. This last assumption explains why in the control peptide treatment group, unlike the 2 other treatment groups, the inverse fluctuations of the CD4^+^CD25^+^Foxp3^+^ and CD4^+^CD25^−^ populations were absent since, as mentioned above, in this group only the level of CD8^+^CD28^−^Foxp3^+^ cells did not demonstrate a substantial and persistent rise.

**Figure 5:**
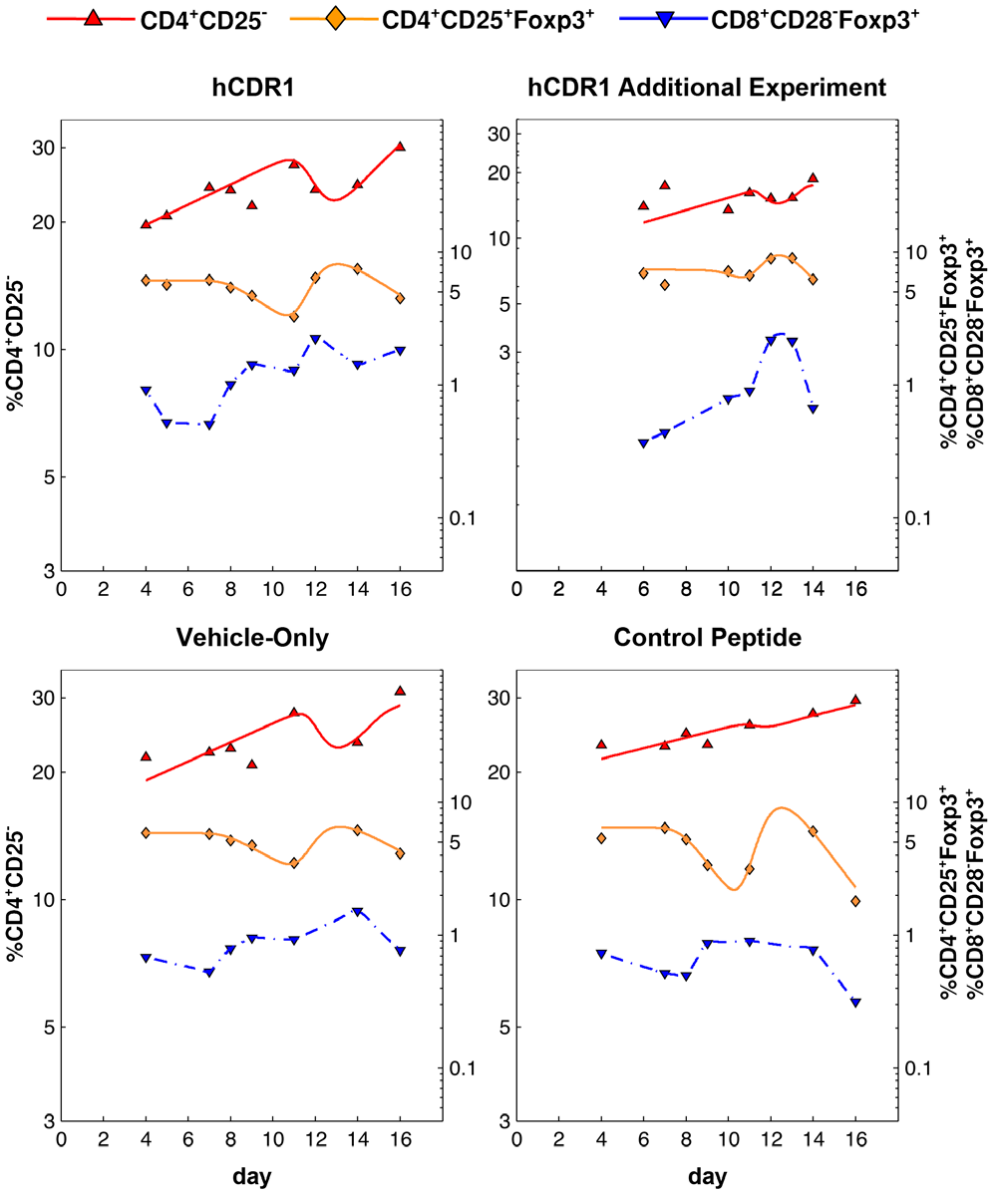
Fitting a mathematical model to the measured data. The plotted solid curves were generated using a mathematical model (see Materials and Methods for details), fitting the measured levels of CD4^+^CD25^−^ (red, left y-axis) and CD4^+^CD25^+^Foxp3^+^ (orange, right y-axis) cells in days 4–16 (days 6–14 for the additional experiment involving the hCDR1 treatment). The approximated levels of CD8^+^CD28^−^Foxp3^+^ cells (blue, dashed line, right y-axis) were not produced by the model, but were rather interpolated from the actual measurements and used as an input to the model. Points represent the daily averages of the values measured by FACS. Note the logarithmic scale of both y-axes.

Figure 5 presents the results of fitting the mathematical model to the kinetics observed in days 4–16 (after the transient initial rise). As can be seen, the predictions generated by the model agree well with the measured levels of cells. Thus, we may conclude that the suggested mechanism is not refuted by the available measurements and explains them well.

We note that in the fitted mathematical model, it is assumed that CD4^+^CD25^−^ cells respond to *changes* in the level of CD4^+^CD25^+^Foxp3^+^ cells, rather than to their absolute level itself. This assumption is justified directly by the kinetic data: as can be seen in Figure 4, for example for the hCDR1-injected group, the level of CD4 Tregs was generally lower between days 11–12, compared to their level between days 14–16; nonetheless, the CD4^+^CD25^−^ subset dropped between days 11 and 12, and rose between days 14 and 16. Thus, the absolute level of CD4 Tregs cannot be used to explain the kinetics of CD4^+^CD25^−^ cells, unlike changes in this level. A similar picture is evident with regard to the vehicle-only treatment group. Accordingly, models which assume dependence between CD4^+^CD25^−^ dynamics and the absolute level of CD4^+^CD25^+^Foxp3^+^ could not produce a good fit to the measured data.

### Conversion Unlikely to Be the Sole Source of CD4 Tregs

The model described above assumes that the CD4^+^CD25^+^Foxp3^+^ population is capable of undergoing expansion, and that this process constitutes the main source for its increase, rather than conversion of CD4^+^CD25^−^ and/or CD4^+^CD25^+^Foxp3^−^ cells. While this assumption yields a good fit to the data for all 3 treatment groups, we cannot rule out the possibility that conversion is the major contributor to the late rise (days 11–14) of CD4 Tregs cells in two of the treatment groups – the mice injected with hCDR1, and those injected with vehicle-alone. However, in the third group – the control peptide-injected mice – conversion cannot play such a role, as in this arm the substantial rise of the CD4^+^CD25^+^Foxp3^+^ population between days 11 and 14 is not accompanied by a concurrent decrease in the CD4^+^CD25^−^ and CD4^+^CD25^+^Foxp3^−^ subsets.

## Discussion

In this work we have combined, for the first time, mathematical modeling with frequent *in vivo* sampling, in order to characterize the processes responsible for the development of regulatory T cells in response to the tolerogenic administration of a peptide. The results obtained taking this approach predicted the emergence of functional CD4 Tregs based on the occurrence of inverse oscillations of the latter and CD4^+^CD25^−^ cells. Furthermore, we demonstrated that at least in the studied system, CD4 Tregs require a sufficiently high level of CD8 Tregs in order to become functional. In addition, our analysis implied that conversion of effector cells is not the major source of CD4 Tregs here.

The beneficial effect of hCDR1 was previously shown to be mediated by the upregulation of a specific population of CD4 Tregs [4]. Accordingly, we have shown here that the administration of this peptide induced a significant, lasting (over two weeks), non-random perturbation of the immune system. Following an initial, transient rise of all of the studied immune populations (which may be interpreted as a response to the injection itself), a significant expansion of CD8^+^CD28^−^Foxp3^+^ cells preceded the occurrence of inverse oscillations of CD4^+^CD25^+^Foxp3^+^ and CD4^+^CD25^−^ cells, such that a rise in the former subset was accompanied by a fall in the latter and vice versa. This kinetic behavior matches the previously reported capability of hCDR1-induced CD4 Tregs to suppress the proliferation of CD4 effector cells [4], and its dependency on the presence of CD8 Tregs [9]. Moreover, when these hypothesized interrelations between the above 3 cell populations were represented mathematically, the predictions generated by the resulting model fitted well the cell levels measured over time, showing that the suggested mechanism can indeed explain the actual data.

Similar kinetic patterns, albeit with lower magnitudes, were observed in mice injected with vehicle alone, including the above-mentioned inverse oscillations; this suggests functional suppression by CD4 Tregs in these mice as well. However, CD4 Tregs taken from vehicle treated mice are known to be ineffective in suppressing autoreactive T cells in SLE-afflicted mice [4]. One possible explanation for this alleged discrepancy may lie in the *specificities* of the upregulated T cells in each case: while the administration of hCDR1 induces the expansion of cells with specificities relevant to the SLE-context, the cells responding to the injection of the vehicle alone probably represent a plethora of naturally-occurring specificities, non-related to SLE. Although the latter will be non-functional in SLE-afflicted mice, *within* the healthy injected mice we can still expect suppression of CD4 effector cells by CD4 Tregs. The induction of a significant perturbation to the immune system by the injection of a vehicle may be a result of a “danger signal” provided by the injection itself. Thus, the effect observed in the vehicle-treated group may represent an example of a normal regulation of the immune system, which involves not only effector T cells but also Tregs to secure proper balance of the quality and magnitude of the response [24].

It has been previously shown [4] that the injection of the control peptide does not yield functional CD4 Tregs. Indeed, as shown here, in the mice treated with this peptide no inverse oscillations of CD4^+^CD25^−^ and CD4^+^CD25^+^Foxp3^+^ cells were recorded. This can be explained by our observation that in this group the CD8^+^CD28^−^Foxp3^+^ cells did not rise to the same level as in the other two treatment groups, and started declining earlier. It should be noted that while the control peptide binds MHC class II with the same avidity as hCDR1, the injection of the two peptides results nevertheless in different immunological effects. Thus, whereas hCDR1 was shown to down-regulate the production of IFN-γ and to up-regulate TGF-β, the control peptide did not inhibit and sometimes rather led to increased production of IFN-γ and did not affect the expression of TGF-β [25]. These differences could partially explain the inability of the control peptide to effectively induce CD8 Tregs, which are required for the development of functional CD4 Tregs.

The above observations suggest that the administration of hCDR1 and, to a lesser degree, of vehicle alone, promote a transition to a “regulatory regime”, involving a rise in both CD4 and CD8 Treg populations, a suppression of CD4 effector cells and, possibly, the conversion of such cells into CD4 Tregs. The administration of the control peptide seems not be sufficient to trigger such a transition. Thus, the rise of CD8 Tregs is, in this case, less significant and transient, and the resulting CD4 Tregs are non-functional. Accordingly, fitting the observed kinetics with mathematical models showed that while in mice injected with hCDR1 or vehicle alone the late rise of CD4 Tregs may be explained (at least in part) by conversion of CD4 effector cell, this is not the case for the control peptide treatment group. However, the similarity in the kinetic profiles of CD4 Tregs in all 3 arms may imply that conversion is not the sole source of CD4 Tregs also in the other two treatment groups.

Although an increase of CD4^+^CD25^+^Foxp3^+^ cells was observed following the administration of the control peptide, these cells did not suppress the proliferation of CD4^+^CD25^−^ cells; this suggests that Foxp3 is not a sufficient marker for a regulatory phenotype, as was previously discussed [26]. Indeed, additional molecules such as TGF-β, CTLA-4, and Bcl-xL were shown to play a key role in the development of hCDR1-induced functional Tregs [4], [9], [27].

In previous studies, hCDR1 was administered in the context of SLE, namely, it was injected either to SLE-afflicted mice [8], young, SLE-prone mice [4], or mice that were immunized with an SLE-inducing anti-DNA antibody that bears a major idiotype, 16/6Id [9], [28]. The injection of the tolerogenic peptide induced CD8 and CD4 Tregs in the treated mice [4], [9]. In this study, however, we analyzed the development of the two subsets of Tregs in naïve, healthy mice that were administered with a single injection of this tolerogenic peptide during a 16-day follow-up period. The cell status in the disease-free context as compared with that in SLE-afflicted mice is significantly different in regard to their degree of activation, the level of apoptosis, and the production of pathogenic cytokines. Hence, the use of naïve mice enabled us to compare the effects of hCDR1 and a control peptide on the induction of CD8 and CD4 Tregs without the background of general activation of the cells.

The model used here assumes that CD4^+^CD25^−^ cells respond to *changes* in the levels of CD4 Tregs, rather than to their absolute numbers; assuming the latter failed to produce a good fit to the data since in different time intervals, comparable levels of CD4 Tregs were accompanied by either a rise or a drop in CD4^+^CD25^−^ cells. One can avoid the reliance on changes (represented mathematically by the time-derivative) by associating the suppression of CD4^+^CD25^−^ cells with a signal (e.g. a cytokine) having the following properties (see Text S1): it stimulates its own production (i.e. participates in a positive feedback loop), it is consumed by CD4^+^CD25^+^Foxp3^+^ cells, and may serve as a growth factor for them. Although the predicted cytokine may be initially secreted by the Tregs, they are not likely to be its major producers. TGF-β is a possible candidate to serve as this signal, as it answers the above description [4], [28], [29]. It should be noted that while even without these modifications the model may be considered too complicated given the amount of available data, simpler or comparably complicated alternative models tested by us (see Text S1) failed to explain the observed kinetics.

The combination of mathematical models and *in vivo* kinetic measurements utilized in the current work has allowed us to gain new insights into the mechanisms governing the function of regulatory T cells, and to generate predictions that can be further tested in future work.

## Materials and Methods

### Ethics Statement

The study has been approved by the Animal Care and Use committee of the Weizmann Institute of Science.

### Mice

Female BALB/c mice were purchased from Harlan (Jerusalem, Israel).

### Synthetic Peptides

A peptide with the following sequence GYYWSWIRQPPGKGEEWIG (hCDR1) based on the CDR1 of the human anti-DNA monoclonal antibody [30] bearing the 16/6Id was synthesized by Polypeptide laboratories (LA, USA) and used in this study. A peptide with scrambled order of the amino acids of the hCDR1, SKGIPQYGGWPWEGWRYEI (‘scrambled peptide’) was synthesized and used as a control. The control peptide binds MHC class II with an avidity similar to that of hCDR1.

### Treatment of Mice

Mice at the age of 2 months were divided into 3 groups (N = 24 mice for the hCDR1-injected group, N = 18 for the other two groups) and treated with a single subcutaneous injection of either hCDR1 (50 µg/mouse) or the control (scrambled) peptide (50 µg/mouse) that were administered in the vehicle Captisol® (Sulfobutylether beta cyclodextrin that has been designed by CyDex to enhance the solubility and stability of drugs). A third group of mice was treated with a single subcutaneous injection of the vehicle only.

### mAbs

The following Abs were used for immunofluorescent staining of cells: Anti-CD4-PE (clone GK1.5), anti-CD4-APC (clone L3T4), anti-CD25-FITC (clone 7D4), anti-CD8-FITC (clone 53-6.7), and their matched isotype controls were obtained from Southern Biotechnology Associates (Birmingham, AL). Anti-CD28-PE (clone 37.51), and its matched isotype controls were purchased from PharMingen (San Diego, CA, USA). Anti-Foxp3-FITC (clone FJK-16s Set) was purchased from eBioscience (San Diego, CA).

### FACS Analysis

Cells were incubated with the relevant Ab and analyzed by FACS (Becton Dickinson, Franklin Lakes, NY). For intracellular staining, the cells were incubated with a fixation solution, washed, and resuspended in permeabilization solution (Serotec; Oxford, UK).

### Kinetic Analysis

The percentages of the 6 different cell populations (CD4^+^CD25^−^, CD4^+^CD25^+^Foxp3^−^, CD4^+^CD25^+^Foxp3^+^, CD8^+^CD28^+^, CD8^+^CD28^−^Foxp3^−^, CD8^+^CD28^−^Foxp3^+^) out of total viable splenocytes, as obtained from FACS measurements, were used for the analysis of kinetics and fitting by mathematical models. Measurements were taken on days 0, 2, 3, 4, 5, 7, 8, 9, 11, 12, 14 and 16 following injection for the mice treated with hCDR1, and on days 0, 2, 3, 4, 7, 8, 9, 11, 14 and 16 following injection for the two other treatment groups. Cell subset percentages, rather than absolute cell numbers, were used in the analysis, due to the additional noise detected in the latter due to differences in spleen sizes and cell numbers between individual mice.

### Mathematical Models

ODE-based mathematical models were used to fit the levels of CD4^+^CD25^−^ cells (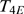) and CD4^+^CD25^+^Foxp3^+^ cells, which were assumed to be either non-functional (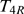) or functional (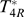). The dynamics of CD8^+^CD28^−^Foxp3^+^ cells (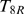) were not modeled here, but were rather interpolated from the data and used as an input to the models. We assumed that cell dynamics in the first few days following the s.c. injection represented a transient response to it. Thus, our models, describing the suppressive effect of Tregs, focused on the dynamics during the second part of the experiment period.

Several different models were tested (see Text S1), but only one model, described next, yielded a fit that was able to describe the entire observed kinetic pattern:
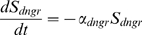
(1)

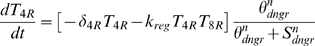
(2)

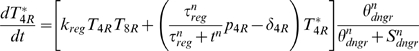
(3)

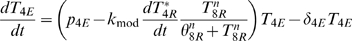
(4)


In this model, 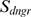 represents a “danger signal”, induced by the initial subcutaneous injection; it is assumed, in the absence of an actual immunogenic signal, that 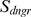 decays exponentially with rate constant 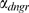. Initially, 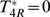. As long as the danger signal is larger than a certain threshold 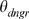, the population of CD4^+^CD25^+^Foxp3^+^ cells remains stable and nonfunctional with an initial level 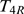 = 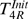. Once the danger signal decays below 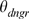 and following a necessary interaction with CD8^+^CD28^−^Foxp3^+^ cells, 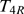 cells start differentiating into functional CD4 Tregs with probability 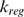 per 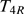-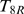-interaction. Both functional and nonfunctional CD4 Tregs are removed from the system with rate 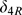. It is assumed that nonfunctional Tregs do not significantly expand, while functional Tregs expand with rate 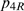, as long as the time since injection does not exceed a certain threshold 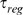. The basic proliferation rate constant of CD4^+^CD25^−^ cells (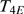) is 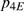, and it is modulated by changes in the level of functional CD4 Tregs with a factor 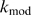. This process also requires that the level of CD8^+^CD28^−^Foxp3^+^ (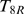) cells is above a certain threshold, 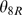. CD4^+^CD25^−^ cells are assumed to be removed from the system with rate 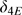. 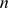 is assumed to be an exponent large enough to produce effective step-functions.

Note that since measurements of the danger signal are not available, we replaced the dependency on this signal (i.e. the Hill functions in Eqs. 2 & 3) by a step function dependent on time. Furthermore, a possible biological explanation for the dependency on the time derivative of 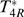 appearing in Eq. 4 is a signal, e.g. cytokine, that is strongly affected by changes in the 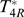 population (see a detailed description of an explicit model with such signal in Text S1). Note also that we do not necessarily assume that all of the CD4^+^CD25^−^ cells respond to the interactions with the functional CD4 Tregs; it is possible to split the population of CD4^+^CD25^−^ cells into “responding” and “non-responding” (static) cells, without affecting the quality of the resulting fit.

### Nonlinear Fitting

Mathematical models were fitted to the experimental data using Berkeley Madonna (University of California, Berkeley, CA). Due to the exploratory nature of this study, hence a limited amount of collected data, the goal of fitting here was to assess the plausibility of qualitatively different models, rather than to estimate the values of parameters. Daily averages (2 mice each day) were fitted rather than individual measurements. The kinetics of the first 4 days of each experiment (6 days for the additional hCDR1 experiment) were not fitted, due to the assumptions described above.

### Statistical Analysis

Within-day variation was estimated by the difference between each measurement and its corresponding daily average, divided by the average. Between-days variation was estimated by considering the differences between daily averages and the corresponding average over time, divided by the latter. The above normalization step was used in order to allow the comparison of all cell subsets together. The two samples of relative differences were then compared using the non-parametric Mann-Whitney U-test.

## Supporting Information

Text S1 Additional mathematical models.
(0.09 MB DOC)

Table S1 The cross-correlation values between the time series of each cell subset, compared across each pair of treatment groups. Two numbers are reported for each case: the cross-correlation value, and the corresponding p-value, i.e. the probability of getting this cross-correlation value by chance. P-values were computed using the following non-parametric procedure: given a pair of time series to be compared, 1,000 different random permutations were generated from one of them, and the cross-correlation value was then computed between the second series and each permutation. The cross-correlation value computed for the original series was then ranked with reference to these 1,000 values, yielding the reported p-value.
(0.03 MB DOC)

Figure S1 The kinetics of CD4^+^CD25^+^Foxp3^+^ cells expressed in absolute cell numbers. Similar patterns to those determined by FACS for percentages of cells were observable (compare to Figure 2E in the text); however, additional noise was detectable in measurements referring to absolute numbers of cells (see text). The legend is the same as in Figure 2.
(0.13 MB TIF)

## References

[1] Sakaguchi S (2004). Naturally arising CD4^+^ regulatory T cells for immunologic self-tolerance and negative control of immune responses.. Annu Rev Immunol.

[2] Lan RY, Ansari AA, Lian ZX, Gershwin ME (2005). Regulatory T cells: development, function and role in autoimmunity.. Autoimmun Rev.

[3] Masteller EL, Warner MR, Ferlin W, Judkowski V, Wilson D (2003). Peptide-MHC class II dimers as therapeutics to modulate antigen-specific T cell responses in autoimmune diabetes.. J Immunol.

[4] Sharabi A, Zinger H, Zborowsky M, Sthoeger ZM, Mozes E (2006). A peptide based on the complementarity-determining region 1 of an autoantibody ameliorates lupus by up-regulating CD4^+^CD25^+^ cells and TGF-beta.. Proc Natl Acad Sci U S A.

[5] Singh RP, La Cava A, Hahn BH (2008). pConsensus peptide induces tolerogenic CD8+ T cells in lupus-prone (NZB×NZW)F1 mice by differentially regulating Foxp3 and PD1 molecules.. J Immunol.

[6] Shevach EM (2006). From vanilla to 28 flavors: multiple varieties of T regulatory cells.. Immunity.

[7] Sthoeger ZM, Dayan M, Tcherniack A, Green L, Toledo S (2003). Modulation of autoreactive responses of peripheral blood lymphocytes of patients with systemic lupus erythematosus by peptides based on human and murine anti-DNA autoantibodies.. Clin Exp Immunol.

[8] Luger D, Dayan M, Zinger H, Liu JP, Mozes E (2004). A peptide based on the complementarity determining region 1 of a human monoclonal autoantibody ameliorates spontaneous and induced lupus manifestations in correlation with cytokine immunomodulation.. J Clin Immunol.

[9] Sharabi A, Mozes E (2008). The suppression of murine lupus by a tolerogenic peptide involves foxp3-expressing CD8 cells that are required for the optimal induction and function of foxp3-expressing CD4 cells.. J Immunol.

[10] Perelson AS (2002). Modelling viral and immune system dynamics.. Nat Rev Immunol.

[11] Borghans JA, de Boer RJ (2007). Quantification of T-cell dynamics: from telomeres to DNA labeling.. Immunol Rev.

[12] Regoes RR, Yates A, Antia R (2007). Mathematical models of cytotoxic T-lymphocyte killing.. Immuno Cell Biol.

[13] Ho DD, Neumann AU, Perelson AS, Chen W, Leonard JM (1995). Rapid turnover of plasma virions and CD4 lymphocytes in HIV-1 infection.. Nature.

[14] Neumann AU, Lam NP, Dahari H, Gretch DR, Wiley TE (1998). Hepatitis C viral dynamics in vivo and the antiviral efficacy of interferon-alpha therapy.. Science.

[15] Jiang Q, Su H, Knudsen G, Helms W, Su L (2006). Delayed functional maturation of natural regulatory T cells in the medulla of postnatal thymus: role of TSLP.. BMC Immunol.

[16] Vukmanovic-Stejic M, Zhang Y, Cook JE, Fletcher JM, McQuaid A (2006). Human CD4^+^ CD25hi Foxp3^+^ regulatory T cells are derived by rapid turnover of memory populations in vivo.. J Clin Invest.

[17] Zelinskyy G, Kraft AR, Schimmer S, Arndt T, Dittmer U (2006). Kinetics of CD8^+^ effector T cell responses and induced CD4^+^ regulatory T cell responses during Friend retrovirus infection.. Eur J Immunol.

[18] Haribhai D, Lin W, Relland LM, Truong N, Williams CB (2007). Regulatory T cells dynamically control the primary immune response to foreign antigen.. J Immunol.

[19] Kim JM, Rasmussen JP, Rudensky AY (2007). Regulatory T cells prevent catastrophic autoimmunity throughout the lifespan of mice.. Nat Immunol.

[20] Mahnke K, Schönfeld K, Fondel S, Ring S, Karakhanova S (2007). Depletion of CD4^+^CD25^+^ human regulatory T cells in vivo: kinetics of Treg depletion and alterations in immune functions in vivo and in vitro.. Int J Cancer.

[21] Selvaraj RK, Geiger TL (2007). A kinetic and dynamic analysis of Foxp3 induced in T cells by TGF-beta.. J Immunol.

[22] Ueha S, Yoneyama H, Hontsu S, Kurachi M, Kitabatake M (2007). CCR7 mediates the migration of Foxp3^+^ regulatory T cells to the paracortical areas of peripheral lymph nodes through high endothelial venules.. J Leukoc Biol.

[23] Nolte-'t Hoen EN, Boot EP, Wagenaar-Hilbers JP, van Bilsen JH, Arkesteijn GJ (2008). Identification and monitoring of effector and regulatory T cells during experimental arthritis based on differential expression of CD25 and CD134.. J Leukoc Biol.

[24] Sakaguchi S, Yamaguchi T, Nomura T, Ono M (2008). Regulatory T cells and immune tolerance.. Cell.

[25] Sharabi A, Haviv A, Zinger H, Dayan M, Mozes E (2006). Amelioration of murine lupus by a peptide, based on the complementarity determining region-1 of an autoantibody as compared to dexamethasone: different effects on cytokines and apoptosis.. Clin Immunol.

[26] Banham AH, Powrie FM, Suri-Payer E (2006). FOXP3+ regulatory T cells: Current controversies and future perspectives.. Eur J Immunol.

[27] Sharabi A, Luger D, Ben-David H, Dayan M, Zinger H (2007). The role of apoptosis in the ameliorating effects of a CDR1-based peptide on lupus manifestations in a mouse model.. J Immunol.

[28] Sela U, Mauermann N, Hershkoviz R, Zinger H, Dayan M (2005). The inhibition of autoreactive T cell functions by a peptide based on the CDR1 of an anti-DNA autoantibody is via TGF-beta-mediated suppression of LFA-1 and CD44 expression and function.. J Immunol.

[29] Yamagiwa S, Gray JD, Hashimoto S, Horwitz DA (2001). A role for TGF-beta in the generation and expansion of CD4+CD25+ regulatory T cells from human peripheral blood.. J Immunol.

[30] Waisman A, Shoenfeld Y, Blank M, Ruiz PJ, Mozes E (1995). The pathogenic human monoclonal anti-DNA that induces experimental systemic lupus erythematosus in mice is encoded by a VH4 segment.. Int Immunol.

