# Supplementary material for: In Vivo Dynamical Interactions between CD4 Tregs, CD8 Tregs and CD4+CD25− Cells in Mice
Source: PLoS One. 2009 Dec 24;4(12):e8447. doi: 10.1371/journal.pone.0008447 (PMC2794381; doi:10.1371/journal.pone.0008447)
Supplement: Text S1 — Additional mathematical models. (0.09 MB DOC) [file pone.0008447.s001.doc]

**Replacing the Dependency on the Time-Derivative of Functional CD4 Tregs by a Dependency on a Signal**

It is possible to introduce into the model described in the text a signal (e.g. a cytokine), $S$, which mediates the modulation of the proliferation of CD4^+^CD25^-^ cells by functional CD4 Tregs. This allows to replace the assumption that the dynamics of the former population are dependent on the time derivative of the latter (see Eq. 4 in the text). Furthermore, by coupling this signal to the expansion term of functional CD4 Tregs, it is possible to explain their dynamics in the second part of the experiment, and in particular their eventual decline, without the need to assume that the entire process ends at a certain time point $\tau_{reg}$.

The equation for $S$ can be derived by setting it proportional to . If we assume that the functional CD4^+^CD25^+^Foxp3^+^ cells undergo logistic growth, the resulting equation has the form

$\frac{dS}{dt}=k_{S}S-a_{S}ST_{4R}^{*}-\delta_{S}S$ (5)

That is: (a) The signal $S$ enhances its own production, through a positive feedback loop; (b) It is consumed by functional CD4^+^CD25^+^Foxp3^+^ cells; (c) It is cleared from the system with a fixed rate.

In order to incorporate the effect of this signal on the proliferation rate of CD4^+^CD25^-^ cells, Eq. 4 in the text is replaced by the equation

$\frac{dT_{4E}}{dt}=\left[p_{4E}-k_{\mathrm{mod}}(S-\theta_{S})\frac{T_{8R}^{n}}{\theta_{8R}^{n}+T_{8R}^{n}}\right]T_{4E}-\delta_{4E}T_{4E}$ (6)

According to this equation, whenever $S$ is higher than a certain threshold - $\theta_{S}$ - the proliferation rate of these cells decreases, and vice versa.

The same signal can be further used to explain the kinetics of the functional CD4^+^CD25^+^Foxp3^+^ cells, by coupling it to the expansion rate of these cells, i.e. by replacing Eq. 3 with the equation

$\frac{dT_{4R}^{*}}{dt}=\left\{k_{reg}T_{4R}T_{8R}+\left[p_{4R}\frac{S}{\theta_{S4R}+S}\left(1-\frac{T_{4R}^{*}}{C_{4R}}\right)-\delta_{4R}\right]T_{4R}^{*}\right\}\frac{\theta_{dngr}^{n}}{\theta_{dngr}^{n}+S_{dngr}^{n}}$ (7)

Thus, $S$ can be thought of as a *growth factor* of the functional CD4^+^CD25^+^Foxp3^+^ cells, consumed by them and stimulating their expansion. This alteration of Eq. 3 allows to drop the dependency on $\tau_{reg}$, as mentioned above.

We note that the equation for $S$ does not necessarily imply that its secretion is not affected by the level of functional CD4^+^CD25^+^Foxp3^+^ cells. It may very well be that these cells initially stimulate the production of $S$, but that this effect is insensitive to their level, once it is higher than some relatively low threshold. That is, Eq. 5 may approximate a more complicated one:

$\frac{dS}{dt}=\left(k_{S}^{'}+k_{S}^{''}\frac{T_{4R}^{*}}{\theta_{4RS}+T_{4R}^{*}}\right)S-a_{S}ST_{4R}^{*}-\delta_{S}S$ (8)

where $\theta_{4RS}$ is relatively small, such that most of the time $T_{4R}^{*}$ is much larger than $\theta_{4RS}$.

Finally, we note that in order to reduce the number of free parameters in this version of model, it is possible to further rely on the relation between $S$ and and to derive the parameters $k_{S}$, $a_{S}$ and $\delta_{S}$ directly from the equation of $T_{4R}^{*}$, without reducing the quality of the fit obtained to the experimental data.

**Additional Mathematical Models**

Below we describe two of the several additional mathematical models tested against the observed kinetics, failing to produce a good fit to the data. In both cases, it is possible to explain this failure by specific properties of the given kinetics; thus, this failure does not stem from a mere inappropriate choice of parameter values. It should be noted that both models presented here, as well as most of the other models considered, were not inherently simpler than the model presented in the paper itself; in particular, they do not involve a smaller number of parameters. Thus, the failure to produce a good fit using them cannot be simply explained by a smaller number of degrees of freedom.

***i. A model assuming suppression dependent on the absolute level of CD4 Tregs***

The actual model used in fitting the data assumes that the proliferation of the CD4^+^CD25^-^ cells is modulated based on *changes* (time-derivative) in the levels of functional CD4 Tregs, rather than their absolute levels. The model described by the following equations assumes the opposite, and differs from the model used in the equation for $T_{4E}$ only:

$\frac{dS_{dngr}}{dt}=-\alpha_{dngr}S_{dngr}$ (9)

$\frac{dT_{4R}}{dt}=\left[-\delta_{4R}T_{4R}-k_{reg}T_{4R}T_{8R}\right]\frac{\theta_{dngr}^{n}}{\theta_{dngr}^{n}+S_{dngr}^{n}}$ (10)

$\frac{dT_{4R}^{*}}{dt}=\left[k_{reg}T_{4R}T_{8R}+\left(\frac{\tau_{reg}^{n}}{\tau_{reg}^{n}+t^{n}}p_{4R}-\delta_{4R}\right)T_{4R}^{*}\right]\frac{\theta_{dngr}^{n}}{\theta_{dngr}^{n}+S_{dngr}^{n}}$ (11)

$\frac{dT_{4E}}{dt}=\left[p_{4E}-k_{\mathrm{mod}}\left(T_{4R}^{*}-\theta_{4R}\right)\frac{T_{8R}^{n}}{\theta_{8R}^{n}+T_{8R}^{n}}\right]T_{4E}-\delta_{4E}T_{4E}$ (12)

Here, if the level of functional CD4 Tregs is larger than a certain threshold $\theta_{4R}$, the proliferation rate of CD4^+^CD25^-^ cells decreases; otherwise, it increases.

The reason for the failure to produce a good fit using this model can be explained using the levels of CD4^+^CD25^+^Foxp3^+^ cells late in the experiment, with regard to the hCDR1 treatment group – see text.

***ii. A model assuming conversion as the sole source of CD4^+^CD25^+^Foxp3^+^ cells***

The next model assumes that the sole source of CD4^+^CD25^+^Foxp3^+^ cells is the conversion of CD4^+^CD25^-^ cells:

$\frac{dS_{dngr}}{dt}=-\alpha_{dngr}S_{dngr}$ (13)

$\frac{dT_{4R}}{dt}=-\delta_{4R}T_{4R}\frac{\theta_{dngr}^{n}}{\theta_{dngr}^{n}+S_{dngr}^{n}}+k_{conv}T_{4E}\frac{\tau_{reg}^{n}}{\tau_{reg}^{n}+t^{n}}\frac{\lambda_{dngr}^{n}}{\lambda_{dngr}^{n}+S_{dngr}^{n}}\frac{T_{8R}^{n}}{\theta_{8R}^{n}+T_{8R}^{n}}$ (14)

$\frac{dT_{4E}}{dt}=p_{4E}T_{4E}-k_{conv}T_{4E}\frac{\tau_{reg}^{n}}{\tau_{reg}^{n}+t^{n}}\frac{\lambda_{dngr}^{n}}{\lambda_{dngr}^{n}+S_{dngr}^{n}}\frac{T_{8R}^{n}}{\theta_{8R}^{n}+T_{8R}^{n}}-\delta_{4E}T_{4E}$ (15)

It is assumed here that this conversion starts only when the danger signal is lower than a certain additional threshold, $\lambda_{dngr}$ ($\lambda_{dngr}<\theta_{dngr}$), and requires a sufficient level of CD8^+^CD28^-^Foxp3^+^ cells. As in the model described in the text, it is assumed that the conversion of CD4^+^CD25^-^ cells into CD4^+^CD25^+^Foxp3^+^ cells is terminated at some time $\tau_{reg}$.

This model, as well as several variations of it (including some assuming two sub-populations of CD4^+^CD25^+^Foxp3^+^ cells), failed to explain the observed kinetics – in particular in the control peptide treatment group, where the late rise of CD4^+^CD25^+^Foxp3^+^ cell is not accompanied by a corresponding drop in the level of CD4^+^CD25^-^ cells.
