# Supplementary material for: In Vivo Dynamical Interactions between CD4 Tregs, CD8 Tregs and CD4+CD25− Cells in Mice
Source: PLoS One. 2009 Dec 24;4(12):e8447. doi: 10.1371/journal.pone.0008447 (PMC2794381; doi:10.1371/journal.pone.0008447)
Supplement: Table S1 — The cross-correlation values between the time series of each cell subset, compared across each pair of treatment groups. Two numbers are reported for each case: the cross-correlation value, and the corresponding p-value, i.e. the probability of getting this cross-correlation value by chance. P-values were computed using the following non-parametric procedure: given a pair of time series to be compared, 1,000 different random permutations were generated from one of them, and the cross-correlation value was then computed between the second series and each permutation. The cross-correlation value computed for the original series was then ranked with reference to these 1,000 values, yielding the reported p-value. (0.03 MB DOC) [file pone.0008447.s002.doc]

| **Population** | **hCDR1 vs.**  **Vehicle-only** | **hCDR1 vs.**  **Control Peptide** | **Vehicle-only vs.**  **Control Peptide** |
| --- | --- | --- | --- |
| **CD4^+^CD25^-^** | 0.94  <0.001 | 0.84  0.001 | 0.91  0.002 |
| **CD4^+^CD25^+^Foxp3^-^** | 0.87  0.001 | 0.87  0.002 | 0.94  <0.001 |
| **CD4^+^CD25^+^Foxp3^+^** | 0.71  0.030 | 0.76  0.009 | 0.81  0.009 |
| **CD8^+^CD28^+^** | 0.68  0.032 | 0.31  0.416 | 0.83  0.001 |
| **CD8^+^CD28^-^Foxp3^-^** | 0.78  0.010 | 0.78  0.010 | 0.85  0.001 |
| **CD8^+^CD28^-^Foxp3^+^** | 0.65  0.035 | 0.41  0.242 | 0.40  0.234 |

**Table S1.** **The cross-correlation values between the time series of each cell subset, compared across each pair of treatment groups.** Two numbers are reported for each case: the cross-correlation value, and the corresponding p-value, i.e. the probability of getting this cross-correlation value by chance. P-values were computed using the following non-parametric procedure: given a pair of time series to be compared, 1,000 different random permutations were generated from one of them, and the cross-correlation value was then computed between the second series and each permutation. The cross-correlation value computed for the original series was then ranked with reference to these 1,000 values, yielding the reported p-value.
