# Supplementary figures and images for: In Vivo Dynamical Interactions between CD4 Tregs, CD8 Tregs and CD4+CD25− Cells in Mice
Source: PLoS One. 2009 Dec 24;4(12):e8447. doi: 10.1371/journal.pone.0008447 (PMC2794381; doi:10.1371/journal.pone.0008447)

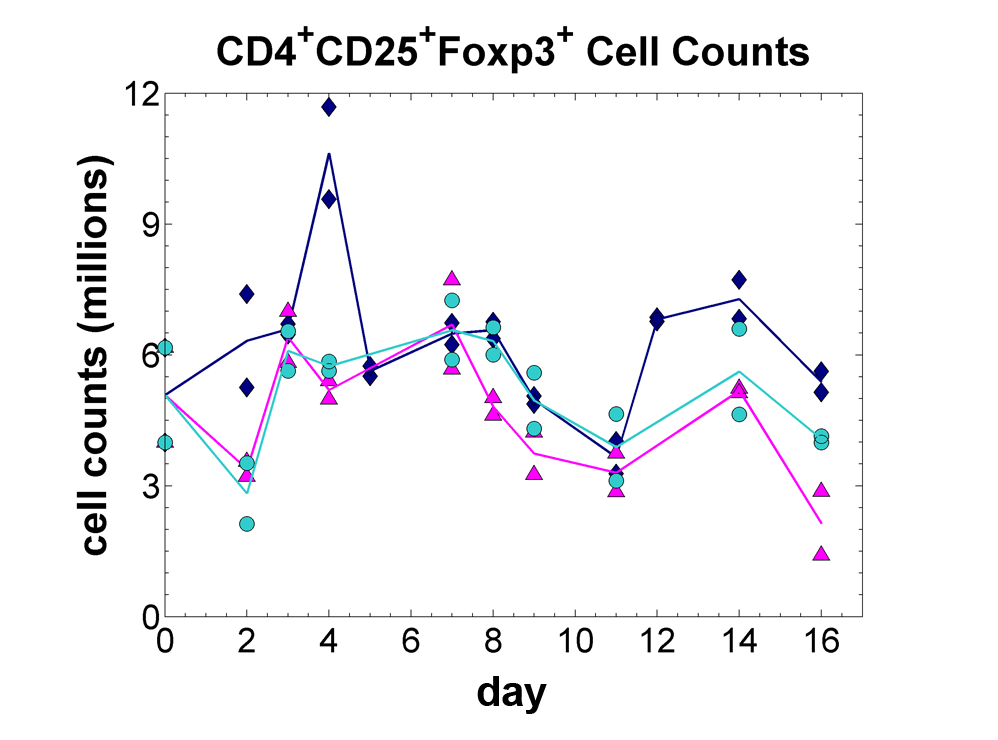

Supplement: Figure S1 — The kinetics of CD4+CD25+Foxp3+ cells expressed in absolute cell numbers. Similar patterns to those determined by FACS for percentages of cells were observable (compare to Figure 2E in the text); however, additional noise was detectable in measurements referring to absolute numbers of cells (see text). The legend is the same as in Figure 2. (0.13 MB TIF) [file pone.0008447.s003.tif]
